# Training for the future: Introducing foundational skills necessary to promote patient-centered care practice in medical physics graduate programs

**DOI:** 10.1016/j.tipsro.2022.09.009

**Published:** 2022-10-01

**Authors:** Laura Padilla, Dina T. Garcia, Anna Rodrigues, Megan Hyun

**Affiliations:** a University of California, San Diego, United States; b Virginia Commonwealth University, One Capital Square 4-114, 830 E. Main Street, P.O. Box 980430, Richmond, VA 23298-0430, United States; c Duke University Medical Center, P.O. Box 3295, Durham, NC 27710, United States; d University of Nebraska Medical Center, 986861 Nebraska Medical Center, Omaha, NE 68198-6861, United States

**Keywords:** Patient-centered care, Medical physics, Effective communication, Critical reflection, Ethics, Graduate education

## Abstract

Current medical physics graduate training in the United States seldom explicitly includes education on foundational skills necessary to produce Patient-Centered Care (PCC)-focused healthcare providers. Such abilities include effective communication, critical reflection, and ethical decision-making. In this article, we present examples of curricula used to purposefully introduce these skills into graduate training to fill this gap. Presented didactic activities include an introduction to patient communication, ethics in medical physics, and a primer in health disparities for medical physicists. Although development of new curricula is resource-intensive when left to individual programs, we here propose resource-sharing and interprofessional collaboration to overcome these barriers.

## Introduction

Patient-Centered Care (PCC), also referred to as Patient and Family-Engaged Care (PFEC), has gained popularity in recent years as it has been shown to improve patient satisfaction and may contribute to better outcomes, including a reduction in racial and ethnic disparities in health care quality [1], [2], [3]. Establishing a ubiquitous culture of PCC across health care systems is vital to ensure that populations have equitable opportunities to achieve and maintain health [4]. Its successful incorporation into clinical practice necessitates, amongst other factors, staff awareness and training. [5], [6], [7] Since medical physicists are an integral part of radiation oncology, their knowledge of PCC practices (or lack thereof) and skills required to accomplish them can impact the implementation of PCC in clinical settings. This is regardless of whether the physicists are patient-facing members of the care team, since PCC is also impacted by factors such as team communication and more patient-centered workflow and process design [6], [7]. Although best practices may evolve, ethical decision-making, effective communication and critical reflection are foundational skills at the heart of PCC [8]. Instruction on effective communication is, at a minimum, embedded into the training of other healthcare professions. [9], [10], [11] This is generally not true for medical physics education in the United States partly because graduate and residency program requirements for such topics are not clearly defined [12], [13]. Any formalized trainings that exist are driven by individual programs [14]. To best contribute to a PCC culture, medical physicists must get exposed to these foundational skills as early as possible in their education so they can have ample time to develop them prior to independent practice and adopt them as part of their professional identity. In this article, we present examples of course units for graduate medical physics programs that can be used to address this gap.

## Examples of curricula to initiate development of foundational patient-centered care skills

We will give a brief overview of three curricula, at different stages of implementation, that introduce effective communication, critical reflection skills, and ethical decision-making to medical physics graduate students through learning about patient communication, ethics, and health disparities. A table summarizing the characteristics of these educational activities (description, pedagogical approach, setting, duration, and evaluation, and outcomes) can be found as a supplemental material.

### Patient communication training

An introductory curriculum for effective patient communication strategies was developed at Virginia Commonwealth University by an interdisciplinary group consisting of a medical physicist, a radiation oncologist, and social workers. We summarize the design and outcomes here, but further details can be found elsewhere [15]. This educational program was also later implemented at Duke University and a summary of results from both institutions is presented herein.

This training was designed for graduate students following experiential learning theory principles [16]. The curriculum assumed no prior experience interacting with patients and minimal knowledge of the radiotherapy workflow. The training focused on effective communication strategies for patient interactions and, following Kolb’s model, had reflection exercises embedded in the design. The effects of the training on confidence and competence regarding effective communication skills were assessed using pre- and post-training surveys, and evaluation of simulated patient interactions. Evaluation of simulated patient interactions was performed both by the standardized patients (actors playing the role of a patient), and by the students. The former was used to obtain an assessment of the participant’s communication skills from a “patient” point of view, while the latter provided self-evaluation information. Although reflection was not the focus of the training and its impact was not measured, it was introduced as a tool to help students process what was being taught and learn from their experience. To familiarize students with radiation oncology from a patient’s perspective, they first watched a patient testimonial and wrote a reflective piece about it using provided prompts. They then attended an introductory presentation to learn some basics about radiation therapy and participated in a brief simulated patient encounter to assess their communication skills baseline. They later attended a lecture on effective patient communication given by social workers. Finally, they had a second patient encounter to practice their new skills, followed by a group debrief facilitated using the PEARLS framework [17]. To conclude the training, they were asked to write a final reflection on their experience.

**Fig. 1:**
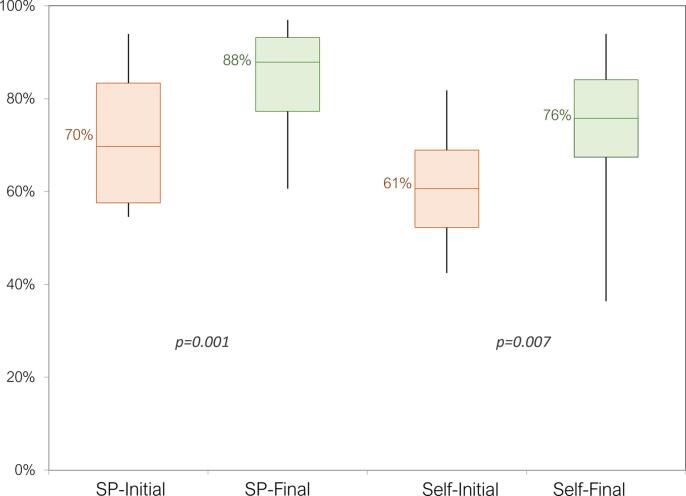
Patient encounter scores for the participants before and after the training, as evaluated by the standardized patient actors (SP-Initial and SP-Final) and by self-evaluation (Self-Initial and Self-Final). There is a statistically significant improvement after the training based on the results of a one-sided Wilcoxon Signed-rank test with an alpha of 0.05 (p = 0.001, and 0.007 for SP, and self-evaluation results, respectively).

**Fig. 2:**
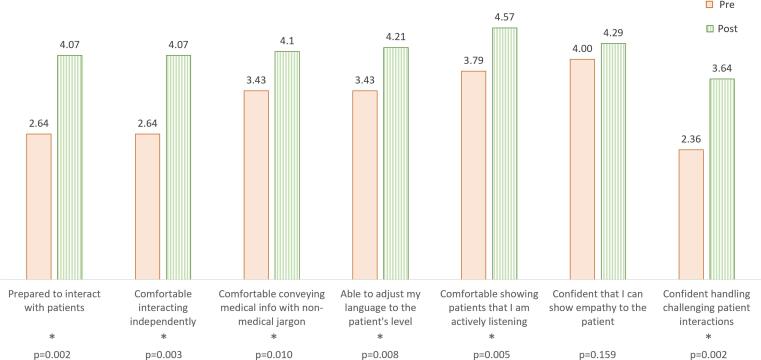
Pre- and post-training survey results. Responses were given based on a 5-point Likert-scale (1 = strongly disagree, 5 = strongly agree). Asterisks denote a statistically significant increase between pre- and post-training results based on a one-sided Wilcoxon Signed-rank test with an alpha of 0.05.

Fourteen participants (8 from VCU and 6 from Duke) completed this program, and their patient interaction scores, as determined by the standardized patients, increased from a median of 70 % to 88 % (Fig. 1). Self-evaluation scores increased from 61 % to 76 % (Fig. 1). These changes are statistically significant (p = 0.001, and 0.007, respectively) based on a one-sided Wilcoxon Signed-Rank test with an alpha of 0.05. The training also significantly improved participants’ self-reported confidence in all domains captured by the survey except for showing empathy (Fig. 2). Although this score improved post-training, the lack of significance is possibly due to its already high pre-activity score. This curriculum has been successfully implemented in two other institutions. Multi-institutional results with both quantitative and qualitative analysis of collected data will be published in the near future.

### Ethics

The basic bioethics principles that have been widely adopted in the field of medical physics (e.g., respect for autonomy, beneficence, nonmaleficence, and justice) are, by definition, patient-centered. [18] It stands to reason that high-quality ethics education, grounded in these principles, is an essential part of patient-centered graduate medical physics education. While Commission on Accreditation of Medical Physics Education Programs (CAMPEP) standards and the American Association of Physicists in Medicine’s (AAPM’s) Task Group 159 (TG 159) report provide guidance on topics that should be covered at the graduate and residency levels[12], [13], [19], the style and breadth of ethics training varies widely between medical physics graduate programs [20]. According to a survey of AAPM members conducted in 2012, “60 % of the respondents (827/1377) stated that they had not received ethics/professionalism education during their medical physics training.” [21]The AAPM has additionally formed a new working group on Ethics Coursework Resources to address the gaps that exist in available ethics training resources [22]. There is clearly a need for ethics education that can prepare medical physics students to exercise critical thinking in their ethical decision-making, so their future careers (whether or not they involve direct patient care) are rooted in patient-centered ethical principles.

We will discuss three teaching tools that are rooted in patient-centered principles and that facilitate this type of critical thinking: (1) case-based learning, (2) interactive eLearning, and (3) timely topical engagement. All three can be incorporated into existing medical physics graduate programs, regardless of whether they offer bioethics training in a dedicated course, as part of a seminar series, or other formats.

Case studies afford students the opportunity to engage with real-world ethical dilemmas using patient-centered ethical principles as a foundation. The use of case studies in ethics education is not a new idea; however, many of the cases available for use in ethics training are broad in their focus and not specific to medical physics. The AAPM’s Working Group on Ethics Case Studies[22] has designed multiple fictional scenarios, with accompanying discussion questions, based on the types of ethical violations that are reported by AAPM members. The first wave of cases developed by this group focused on conflict of interest, social media, and education topics, and have been piloted at the AAPM annual meeting, spring clinical meeting, and in a mixed group of graduate students and residents. These types of case studies allow students to do practice-based learning, which is essential for higher-order thinking skills [23], [24].

Incorporating interactive eLearning modules into ethics and professionalism training facilitates active learning (through both the in-module interaction and allowing flipped-classroom approaches), responds to the shift in how the current generation of digitally native students learns, and allows curriculum to be shared easily between programs, potentially elevating the ethical, patient-centered practice level of all medical physics students [24], [25]. While we see great potential for interactive eLearning modules that focus entirely on medical-physics-specific ethics content, it is also possible to weave ethics content into modules that teach other material as well. In fact, this “interleaving” of different concepts and skills has been shown to be an effective teaching technique for real-world problem solving [24]. The module, “A physicist’s primer for effective patient communication,” developed at the University of Nebraska Medical Center, was designed this way, incorporating interactive slides on the ethics of physicist-patient consultations [26]. A pilot is underway investigating this module’s efficacy for learners at multiple levels, including graduate students. In a preliminary cohort of seven medical physics graduate students at Creighton University, pre- and post-module surveys asked the question, “How important is it to offer physicist-patient consultations in the clinic?” using a modified Likert scale (1–10). The median response increased from 8 to 10 from the pre- to the post-module test, respectively, and this was statistically significant (p = 0.03) using a one-sided Wilcoxon signed-rank test with an alpha of 0.05. While the surveys did not assess the students’ comprehension of ethics concepts directly, this result indicates that the module’s ethical justification of physicist-patient consultations had a significant impact on the learners’ perception of how important these consultations may be.

Finally, ethics curriculum should include engagement with timely topics oriented toward the future of the medical physics field. Two examples of timely topics include the changing role of the medical physicist and dilemmas arising from the use of artificial intelligence (AI) in medicine [27], [28]. These two topics have been presented in graduate medical physics seminars at Creighton University and the University of Wisconsin – Madison, with discussion and exercises built in for learner engagement. There are myriad other ways topics like these could be incorporated into ethics curricula, including case studies and eLearning modules. By allowing students to apply patient-centered ethical principles to new and emerging dilemmas (some of which do not have a widely accepted “right answer”), students are able to exercise critical thinking and practice patient-centered moral reasoning that is relevant to their future careers.

### Health disparities training

The training briefly presented here is the first health disparities (HD) training geared towards medical physicists, to the authors’ knowledge. This curriculum was developed at Virginia Commonwealth University through collaboration between a health disparities scholar and a medical physicist. It was designed following transformative learning theory [29] and its goal was to introduce medical physics graduate students to HD concepts and how they relate to their profession. Attendance was voluntary. The training was divided into four weekly 1.5-hour synchronous online sessions focusing on each of the following objectives: (1) explaining how structural and institutional racism and social determinants of equity contribute to disparities in cancer and access to treatment, (2) examining mistrust, bias, and stereotyping using radiation oncology as an example, (3) encouraging students to identify their role in adopting or developing strategies to address disparities in patient care within the field, and (4) identifying how their knowledge of disparities in cancer and social determinants of equity can help better design public outreach initiatives to combat misinformation about radiation. Sessions consisted of didactic lectures, case studies, large and small group discussions, and reflection exercises, all delivered through Zoom. The impact of the overall training and individual sessions was measured using pre- and post-training surveys comprised of open-ended, categorical, and Likert scale questions based on the course learning outcomes. Questions were designed to assess the impact of the training on participant’s HD knowledge and attitudes. Pre- and post-training surveys were evaluated using descriptive statistics and thematic analysis of open-ended questions. Fifteen trainees attended at least part of the course, with 8–11 participants in each session. Survey response rates varied for the four sessions and full sets of pre- and post-session surveys were collected for 7, 10, 8, and 5 participants, in sessions 1–4, respectively. Most participants reported that weekly sessions increased their feelings of competence to explain the relevance of HD to their role in medical physics (4/7), address mistrust, bias, and stereotyping during patient-provider encounters (6/10), engage in critical reflection (7/8), and design public engagement strategies to reduce HD (5/5). Among participants that completed a pre-post survey for the overall course (N = 4), 75 % reported they will likely/very likely explore issues related to HD in their future education, research, and/or practice. All would recommend this course to colleagues noting satisfaction with topics, atmosphere to discuss sensitive issues, virtual format, activities, and facilitators. This pilot curriculum was successfully delivered based on survey results, but further study is needed with a larger participant pool to evaluate its effectiveness more thoroughly.

## Discussion and conclusions

We have presented here a few examples of curricula that can be implemented at the graduate level to introduce future medical physicists to formalized training promoting effective communication, ethical decision-making, and critical reflection skills. Mastering these talents lays the foundation for patient-centered care practices and provides more holistic training for a profession that is increasingly reliant not only on technical knowledge but also on interpersonal abilities. In a recent study, it was shown that physics consults with patients undergoing radiotherapy reduced anxiety and increased patient satisfaction [30]. In such scenarios, the value of robust training in effective communication and reflective practice, especially early in one’s career, is indisputably an asset. Furthermore, these skills are versatile, and their value surpasses direct patient care. Fostering them enhances interpersonal communication, analytical thinking, and curiosity, which can improve the performance of physicists in and outside of the clinic. Similarly, when holistic training includes an emphasis on applying patient-centered ethical principles to real-world moral dilemmas, students engage in higher-order thinking and practice the type of moral reasoning they are likely to face in their careers. These skills are essential for training patient-centered medical physicists, regardless of whether they work in industry, academia, or clinical settings. Additionally, exposure to holistic training will position medical physics trainees to enter the workforce with a broader understanding of what high-quality healthcare is and how healthcare disparities, social determinants of equity, and PCC impact it. These efforts should move us towards equity by giving rise to a critically reflective medical physics workforce that is committed to fostering a patient-centered culture and open to analyzing and questioning the established systems they encounter.

Implementing these programs is not without challenge. Finding room to fit new material into an already dense curriculum is difficult, and it can become an impossible request if it requires programs to create didactic activities from the ground up. Using short but effective educational activities to introduce these materials, such as the course units presented here, can logistically facilitate the inclusion of these topics. Additionally, practice of these skills could be integrated into other areas already existent in traditional medical physics curricula to provide students with opportunities to practice and further develop these abilities throughout their graduate education. For example, the application of these skills could be embedded into assignments in diagnostic and therapy physics courses, clinical rotations, seminar series, etc and could be made an integral part of research project design and dissemination. Moreover, resource-sharing (i.e. sharing curricula and educational materials across different institutions) could be used amongst medical physics graduate programs to avoid burdening each individual institution with the creation of new trainings. This approach has previously been successfully used to implement trainings in radiation oncology [31]. This, along with forming interprofessional collaborations to develop curricula in areas outside of traditional medical physics expertise, can help alleviate development and implementation issues and make the inclusion of these new skills into our graduate programs more feasible. Additionally, the widespread restructuring of curricula to online modalities during the COVID-19 pandemic serves as precedent that the implementation of (a)synchronous virtual trainings may also be a viable option. Future research efforts should focus on facilitators and barriers to resource-sharing and scaling up implementation of these curricula.

## Declaration of Competing Interest

The authors declare the following financial interests/personal relationships which may be considered as potential competing interests: Funding for the Patient Communication training was provided by Virginia Commonwealth University School of Medicine for VCU students and from Duke University Graduate School for Duke students. Funding for the eLearning project was provided by the University of Nebraska Medical Center Office of Academic Affairs eLearning funded awards program.
