# Supplementary material for: Training for the future: Introducing foundational skills necessary to promote patient-centered care practice in medical physics graduate programs
Source: Tech Innov Patient Support Radiat Oncol. 2022 Oct 1;24:54–8. doi: 10.1016/j.tipsro.2022.09.009 (PMC9547290; doi:10.1016/j.tipsro.2022.09.009)
Supplement: Supplementary data 1 [file mmc1.docx]

**Supplemental Material**

**Table I**. Patient Centered Care training curricula and pedagogical methods.

| **Training** | **Training description and audience** | **Pedagogical approach** | **Setting, duration,**  **and evaluation** | **Outcomes** |
| --- | --- | --- | --- | --- |
| Patient communication training | The goal of this training is to provide an introductory training to effective patient communication skills for medical physics graduate students. | Experiential Learning Theory; Didactic lecture, simulated patient-interaction exercises with individual and group debriefing, reflection exercises | **Setting:** Online via Zoom  **Duration:** Curriculum given over 1.5 days with four distinct portions: Preparation (patient testimonial, introductory presentation <1hr, first simulated patient encounter <15min/participant), instruction (didactic lecture <2hrs), application (second simulated patient encounter <15min/participant), and final reflection  **Evaluation:** pre-post surveys for self-reported confidence changes in communication, and pre-post simulated patient encounter evaluations using a standardized rubric completed by both simulation center actors playing the role of patients and training participants. Statistical significance was determined using a one-sided Wilcoxon sign-ranked test with an alpha of 0.05. | Fourteen graduate students completed the training and all evaluation materials (8 from VCU, 6 from Duke). Participants showed significant improvements both in their confidence (preparedness for patient interaction p=0.002, comfort interacting independently p=0.003, using non-medical jargon p=0.010, adjusting language to patient’s level p=0.008, showing active listening p=0.005, and handling challenging patient interactions p=0.002) and competence (median score given by standardized patient actors increased from 70% to 88%, p=0.001) after the training as evidenced by the survey results and the simulated patient encounters. |
| Incorporating ethics into interactive eLearning | The goal of this initiative is to interleave ethics concepts into other medical physics training (in this case, patient communication training) in an interactive eLearning platform designed for multiple learning levels (graduate students, residents, and practicing physicists). The didactic portion of the module presented a bioethics framework along with a moral justification of physicist-patient communication from the principle of respect for autonomy. | Active and practice-based learning, interleaving; Interactive eLearning module with review questions, branched-scenario simulation, and built-in summative assessment. | **Setting:** eLearning module available online  **Duration:** 20-30 minutes  **Evaluation**: Pre- and post-module surveys assessed both the confidence level of the learners in interacting with patients and the level of agreement with statements on the importance of physicist-patient consultations. Significance was evaluated using a one-sided Wilcoxon signed rank test with an alpha of 0.05. | Seven medical physics graduate students at Creighton University completed the module along with pre- and post-module surveys in 2022. When asked “How important is it to offer physicist-patient consultations in the clinic?” using a modified Likert scale (1-10), the median response increased from 8 to 10 from the pre- to the post-module test, respectively, and this was statistically significant (p=0.03). |
| Health disparities (HD) training implemented at Virginia Commonwealth University | The goal of this pilot curriculum is to introduce medical physics graduate students to HD concepts. Session topics included social determinants of equity, structural racism, implicit bias, public outreach, and critical reflection. | Transformative Learning Theory; Didactic lecture, group discussion, case studies, reflection exercises | **Setting:** Online via Zoom  **Duration:** Four synchronous sessions each 1.5 hours in length  **Evaluation:** pre-post surveys to assess the impact of the training on participant’s HD knowledge and attitudes. Survey questions were developed based on course learning outcomes. Descriptive statistics were generated to compare pre- and post-survey scores. | Fifteen trainees attended at least part of the course with 8-11 attendees/session. Most participants reported that weekly sessions increased their feelings of competence to explain the relevance of HD to their role in medical physics (4/7), address mistrust, bias, and stereotyping during patient-provider encounters (6/10), engage in critical reflection (7/8), and design public engagement strategies to reduce HD (5/5).  Among participants that completed a pre-post survey for the overall course (N=4), 75% reported they will likely/very likely explore issues related to HD in their future education, research, and/or practice. All would recommend this course to colleagues noting satisfaction with topics, atmosphere to discuss sensitive issues, virtual format, activities, and facilitators. |
